# Human Anatomy, Including Structure and Development and Practical Considerations

**Published:** 1907-10

**Authors:** 


HUMAN ANATOMY, INCLUDING STRUCTURE AND DEVELOPMENT AND PRACTICAL CONSIDERATIONS. By Thomas Dwight, M. D., LL. D., Parkman Professor of Anatomy in Harvard University; J. Playfair McMurrich, Ph. D., Professor of Anatomy in the University of Michigan; Carl A. Hamann, M. D., Professor of Anatomy in Western Reserve University; Geo. A. Piersol, M. D., Sc. D., Professor of Anatomy in the University of Pennsylvania; J. William White, M. D., Ph. D., LL. D.; John Rhea Barton, Professor of Surgery in the University of Pennsylvania, with seventeen hundred and thirty-four illustrations, of which fifteen hundred and twenty-two are original, and largely from dissections by John C. Heisler, M. D., Professor of Anatomy in the Medico-Chirurgical College. Edited by Geo. A. Piersol. Philadelphia and London, 1907. J. B. Lippincott Company. Cloth, 2050 pages. Price, $7.50.

This is a magnificent production. It is anatomy rewritten and reillustrated,—not a copy of Gray, which for fifty years has been the foundation of all books on the subject. It is about perfection, both as a text-book and a specimen of the bookmaker's art. That it will have a tremendous sale, and supplant all other anatomies for a half century is certain; there is no room for improvement in any respect. It should be of unusual interest to Americans, because it is the first entirely American anatomy in both text and illustrations. We feel that it marks an important event in American medicine and medical publishing.

It is entirely original in its descriptive matter, its arrangement and its illustrations, for which original dissections were made throughout. It is, consequently, the first all-American anatomy, and, we believe, destined to be a monument to the American medical profession and American publishing. It is doubtful if any work heretofore has had expended upon it the amount of labor and money involved in its production. We cordially commend it.

